# A Pilot Study of the Humoral Response Against the AntiSense Protein (ASP) in HIV-1-Infected Patients

**DOI:** 10.3389/fmicb.2020.00020

**Published:** 2020-01-24

**Authors:** Juliette Savoret, Nathalie Chazal, Jean-Pierre Moles, Edouard Tuaillon, Faroudy Boufassa, Laurence Meyer, Camille Lecuroux, Olivier Lambotte, Philippe Van De Perre, Jean-Michel Mesnard, Antoine Gross

**Affiliations:** ^1^IRIM, Université de Montpellier, CNRS, Montpellier, France; ^2^Pathogenesis and Control of Chronic Infections, INSERM, Etablissement Français du Sang, University of Montpellier, Montpellier, France; ^3^Pathogenesis and Control of Chronic Infections, INSERM, Etablissement Français du Sang, University of Montpellier, CHU Montpellier, Montpellier, France; ^4^INSERM CESP U1018, Université Paris-Sud, Le Kremlin-Bicêtre, France; ^5^IMVA, INSERM, Université Paris-Sud,Le Kremlin-Bicêtre, France; ^6^Department of Internal Medicine and Clinical Immunology, Bicêtre University Hospital, Le Kremlin-Bicêtre, France; ^7^INSERM, CEA UMR 1184, Université Paris-Sud, Le Kremlin-Bicêtre, France

**Keywords:** HIV-1, antisense protein, antibodies, luciferase immuno-precipitation system, viremia

## Abstract

The existence of an antisense Open Reading Frame (ORF) that encodes a putative AntiSense Protein (ASP) on the proviral genome of Human Immunodeficiency Virus type 1 (HIV-1) was a source of debate for 30 years. During the last years, some progresses have been made to characterize the cellular immune response against ASP in HIV-1 seropositive patients. However, no tools were available for the detection of antibodies to ASP in the plasma of HIV-1-infected patients during the natural course of the infection. The aim of our study was to develop a Luciferase Immuno-Precipitation System (LIPS) to monitor the quantitative detection of ASP-specific antibodies in the plasma of HIV-1-infected patients [antiretroviral therapy (ART) naive-patients, patients under ART and HIV-1 controllers], patients who discontinued antiretroviral drugs (ARV). We further used this approach to delineate the epitopes of ASP targeted by antibodies. Antibodies directed against ASP were detected in 3 out of 19 patients who discontinued ARV (15%) and in 1 out of 10 ART-naive patients (10%), but were neither detected in HIV-1 infected patients under ART nor in HIV-1 controllers. Individual variations in levels of ASP-specific antibodies were detected overtime. Both the conserved prolin-rich motif and the core 60–189 region of ASP were found to be essential for antibody recognition in the four patients tested positive for anti-ASP antibodies, who were all untreated at the time of sampling. Moreover, for two of these patients, increased levels of ASP-specific antibodies were observed concomitantly to viremia declines. Overall, our method may represent a useful tool to detect a humoral response to ASP in HIV-1-infected patients, which allowed us to confirm the expression of ASP during the course of HIV-1 infection. Further studies will be needed to fully characterize the humoral response to ASP in HIV-1-infected patients.

## Introduction

The existence of a conserved Open Reading Frame (ORF) located on the antisense strand of the proviral genome of the Human Immunodeficiency Virus type 1 (HIV-1) was first suggested in 1988 (Miller, 1988). In this pioneer study, the antisense ORF was proposed to encode for a highly hydrophobic protein of 189 amino acids, later named AntiSense Protein (ASP). The very first detection of ASP in HIV-1-infected cells was achieved by the group of C. Vaquero in 2002 (Briquet and Vaquero, 2002). The same year, our group demonstrated the existence of Human-T-leukemia Virus Type 1 (HTLV-1) B-ZIP Factor (HBZ), another antisense retroviral protein (Gaudray et al., 2002). Following this discovery, we and others have investigated the antisense transcriptional activity of HIV-1 (Michael et al., 1994; Landry et al., 2007; Laverdure et al., 2012; Arpin-André et al., 2014) and studied ASP both *in vitro* and *ex vivo*. In collaboration with others, we thus successively reported (i) the detection of ASP at the plasma membrane of transfected cells (Clerc et al., 2011), (ii) the involvement of ASP in the autophagic flux of ASP-expressing cells (Torresilla et al., 2013) and importantly, (iii) the detection of a cytotoxic T CD8+ response directed against several domains of ASP in HIV-1 infected patients (Bet et al., 2015). In addition, our group recently showed the concomitant emergence of the 10th gene of HIV-1, the *asp* gene, with the pandemic group M of HIV-1, as well as the existence of a selection pressure acting to maintain *asp* within this group (Cassan et al., 2016). Altogether, these findings brought us to argue that ASP might be involved in the spreading or in the virulence of HIV-1. However, the role endorsed by ASP in the pathophysiology of HIV-1 infection and its underlying cellular mechanisms remain to be unraveled. One way to demonstrate the *in vivo* expression and immunogenicity of ASP, and to assess when ASP is expressed during the course of HIV-1 infection consists in detecting the humoral response elicited against ASP in infected patients. The study from the group of C. Vaquero conducted in 1995 previously revealed by western blot an *in vitro* translated ASP after immuno-precipitation by several serum samples from HIV-1-infected individuals, suggesting the existence of antibodies targeting ASP *in vivo* (Vanhée-Brossollet et al., 1995). However, these promising results have never been reproduced nor confirmed by any other study. In the present study, we developed a specific and quantitative luminescent assay to detect antibodies targeting ASP in a panel of plasma samples from HIV-1-infected patients under antiretroviral therapy (ART), ART-naive or who discontinued antiretroviral drugs (ARV). The LIPS assay presents several advantages. First, the recombinant proteins used in LIPS are obtained from a soluble crude cell lysate extracted from transfected cells, which prevents complex purification protocols. Second, the method used to obtain the recombinant antigens allows the use of native, non-reducing conditions for the antigens. Third, LIPS is a fluid phase immunoassay using antigens in their native conformation and is well suited to detect antibodies directed against linear and conformational epitopes (Burbelo et al., 2015). In the patients tested positive for ASP-specific antibodies, we further delineated the domains of ASP targeted by the antibodies by using ASP deletion mutants. In conclusion, in this study, we provided evidence of the existence of antibodies directed against ASP *in vivo*, we gave insights into the targeted epitopes of ASP, and, for the first time, we described the humoral response against ASP in the plasma of HIV-1-infected individuals.

## Materials and Methods

### Human Plasma Samples

#### ANRS 12174 Trial

Subjects were HIV-1-infected breastfeeding women from Burkina Faso whose infants were enrolled in the PROMISE-PEP trial. All mothers received AZT during pregnancy and labor but they were not eligible for ART post-partum according to national recommendations at the period the trial was conducted (above 350 CD4 cell/μL of blood) (Nagot et al., 2016). Written informed consents were obtained from the mother or the legal representative prior to enrollment (NCT00640263). Plasma samples were screened at week 38 post-partum and at intermediate time points when necessary. The study was conducted according to the sponsor (Agence Nationale de Recherche sur le Sida et les hépatites virales; ANRS) ethic charter, Good Clinical Practices and the principles of the Helsinki declaration. The protocol was approved by the National Ethic Committee (n° 2008-0039).

#### ANRS CO2 SEROCO Cohort

Subjects, enrolled from 1988 to 1995 (i) were infected no more than 12 months prior to enrollment and (ii) were free of AIDS related diseases at inclusion (Hubert et al., 2000). No ART were received before inclusion and during the study until 1996. A follow-up was performed every 6 months until 2009. A written informed consent was obtained from patients, in line with the guidelines formulated by local ethical committees.

#### ANRS HIV Controller CO21 CODEX Cohort

Enrolled subjects met the following criteria: (i) naive for ART, (ii) seropositive for at least 5 years before enrollment, and (iii) with a viral load below 400 HIV-1 RNA copies/mL in the five consecutive measurements before inclusion (Lambotte et al., 2005; Sáez-Cirión et al., 2007) Follow-up was performed at least once a year for 6 years. All patients gave a written informed consent. The C021 CODEX cohort was approved by the Ethics Review Committee of Ile de France VII and performed in compliance with the tenets of the Declaration of Helsinki.

#### Patients Under ART

We used plasma isolated from total blood samples collected from 20 patients under ART during their follow-up at the University Hospital of Montpellier. Subjects enrolled in the cohort provided written informed consent. The study was then approved by Institutional Ethics Committee (RCB n° 2011-A01566-35, AC-2011-1405).

#### Uninfected Donors

Plasma samples were collected from French seronegative blood donors (Etablissement Français du Sang) and from breastfeeding, seronegative women from Zambia. The study was approved by the Excellency in Research Ethics and Science Converge Institutional Review Board (00005948 IRB number) in Lusaka, Zambia.

### Antigens Preparation

Flag-nanoluciferase cDNA (kindly gifted by Dr. Olivier Moncorgé, IRIM) was fused with the codon-optimized cDNA sequence of full-length ASP that has been previously described (Torresilla et al., 2013), or with ASP deletion mutants. The sequences of nano-ASP and of the 26–189 and 60–189 mutants were generated by overlapping PCR from the nanoluciferase and ASP cDNA sequences. For nano-ASP, we used the following primers: nanoluciferase forward 5′-ACGTGAATTCGCC GCCATGGACTACAAGGACGACGATGACAAGGTCTTCACA CTCGAAG-3′ and reverse 5′-CACGGAATTCAGCTCGAGC CGCTGGAGCCCGCCAGAATGCGTTC-3′, ASP forward 5′-GAACGCATTCTGGCGGGCTCCAGCGGCTCGAGTCCCCA GACCGTGAG-3′ and reverse 5′-ACTGCGAATTCACTGCA GCTCCACGCAGGAGTTCAGCAGCACCTGATTCAGCAGT GA-3′. For the 26–189 mutant, the following primer sets were used: nanoluciferase forward 5′-ACGTGAATTCGCCGCCA TGGACTACAAGGACGACGATGACAAGGTCTTCACACTCG AAG-3′ and reverse 5′-GTTATCGGGACTCGAGCCGCTGGAG CCCGCCAGAATGCGTTC-3′, ASP forward 5′-TCCAGCGGC TCGAGTCCCGATAACAACTGCCTG-3′ and reverse 5′-ACTGCGAATTCACTGCAGCTCCACGCAGGAGTTCAGCA GCACCTGATTCAGCAGTGA-3′. For the 60–189 mutant, the following primer sets were used: nanoluciferase forward 5′-ACGTGAATTCGCCGCCATGGACTACAAGGACGACGATGA CAAGGTCTTCACACTCGAAG-3′ and reverse 5′- CAGGGC GGTACTCGAGCCGCTGGAGCCCGCCAGAATGCGTTC-3′; ASP forward 5′-TCCAGCGGCTCGAGTACCGCCCTGTTTT CTCTGTGC-3′ and reverse 5′-ACTGCGAATTCACTGCAGC TCCACGCAGGAGTTCAGCAGCACCTGATTCACAGTGA-3′. The 1–62 and 1–141 nano-ASP deletion mutants were generated from the full-length cDNA sequence of nano-ASP with the forward primer 5′-ACGTGAATTCG CCGCCATGGACTACAAGGACGACGATGACAAGGTCTTCA CACTCGAAG-3′ and the reverse primer 5′-ACTGCGAATTCA GGCGGTGGGGATGGGGGCCTTGTTCCT-3′ (1–62 mutant) or 5′-ATGCTTGAATTCTACGATGTA GGCGCCTC-3′ (1–141 mutant). The coding sequences of nano-ASP and nano-Asp mutants were inserted into p-CAGGS, a mammalian expression vector. Vectors encoding for each construction were transfected into HEK 293T cells using polyethyleneimine (Polysciences Inc.) for 48 h before cell harvest and lysis.

### Luciferase Immuno-Precipitation System

Luciferase Immuno-Precipitation System (LIPS) assay was performed following the protocol described by Burbelo et al. (2015) except for the washing steps which were performed by centrifugation. Briefly, plasma samples were incubated with nano-ASP-containing cell lysates. Immune complexes were then precipitated by adding A/G coated sepharose beads (Thermo Scientific) and the luminescence was revealed by adding the substrate of the nanoluciferase (PROMEGA) and quantified on a Berthold luminescence reader. Plasma samples were added at a final dilution of 1/100 in the reaction. For all LIPS reactions, we initially added the amount of nano-ASP-containing cell lysates required to get a luminescence signal of 1.10^7^ light units. The signal obtained from all LIPS reactions (except for the LIPS reactions using ASP mutants) was normalized according to a calibration standard. This standard consisted in luminescent values obtained by performing LIPS reactions using several dilutions of an anti-FLAG antibody (Bethyl) able to precipitate nano-ASP. In experiments using ASP mutants, the signal was normalized by substracting the signal obtained with the nanoluciferase alone for the same plasma sample. Normalized luminescent signal is expressed as Relative Light Units (RLU). The monoclonal antibody against ASP used to test the LIPS was produced by EUROGENTEC from mice immunized with an HXB2-derived peptide.

### Statistics

The cut-off for positive anti-ASP antibody response was computed from luminescent values obtained with plasma from the nine Zambian and the 20 French uninfected individuals (Z score = 4, which corresponds to a luminescent value of 48,000 RLU).

## Results

### Development of a Quantitative Assay to Detect Antibodies Against ASP in the Plasma of HIV-1-Infected Patients

The LIPS allows for the detection of specific antibodies elicited against pathogens in patients (Burbelo et al., 2014, 2015). In this assay, a luciferase is fused to the antigen of interest, and plasma samples are incubated with cell lysates containing this fusion protein. After an immuno-precipitation step, the presence of antigen-specific antibodies in the plasma of patients is revealed by the luminescence signal emitted by the luciferase. To detect anti-ASP antibodies in the plasma of HIV-1 infected patients, we used a codon-optimized ASP derived from pNL4-3 fused to the nano-luciferase protein (nano-ASP). We first tested the assay with a monoclonal antibody targeting the residues 47 to 62 of ASP, and we obtained a luminescent signal proportional to the concentration of the anti-ASP monoclonal antibody added to the reaction, while no specific signal was obtained when using the nano-luciferase protein alone (Supplementary Figure S1). Therefore, we developed an assay that allows for the specific and quantitative immuno-precipitation of nano-ASP. Using LIPS assay, we then evaluated the presence of specific anti-ASP antibodies in the plasma samples of HIV-1-infected patients.

### Antibodies to ASP Are Detected in Untreated HIV-1-Infected Patients

**FIGURE 1:**
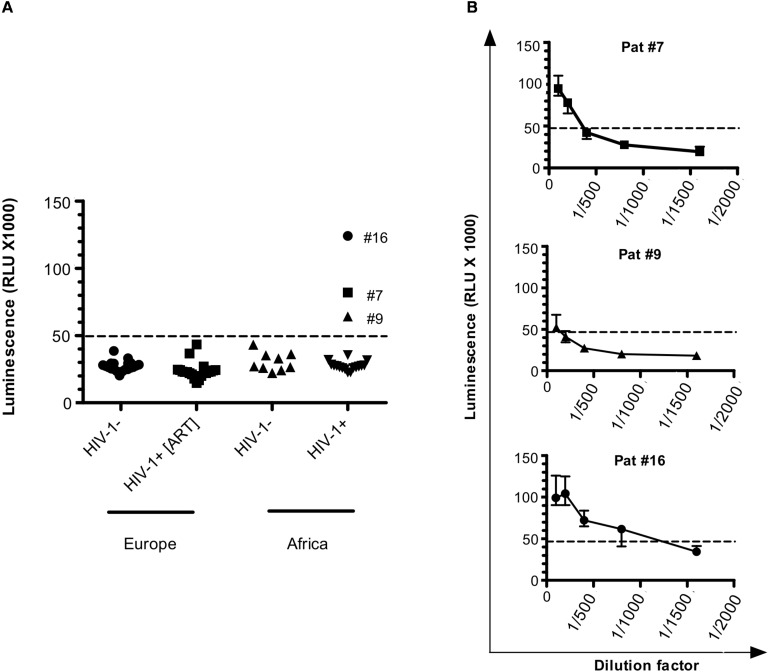
Antibodies against ASP are detected in HIV-1-infected patients. **(A)** Anti-ASP LIPS was conducted on the plasma of HIV-1 infected-patients from two groups: one group of patients from Europe under ART (HIV+ [ART]; *N* = 20) and one group of patients from Africa cleared of any ARV for 37 weeks (HIV+; *N* = 19) (ANRS12174 trial). LIPS was also conducted on the plasma of patients from two seronegative control groups originating from France (*N* = 20) and Zambia (*N* = 9). Signal is expressed as Relative Light Units (RLU). Data shown are the mean of the median of three independent experiments performed in triplicates. **(B)** Plasma samples for Pat #7, Pat #9, and Pat #16, were serially diluted and LIPS was performed on each dilution. Signal is expressed as RLU. Data shown represent the median (plus min and max values) of one representative experiment performed in triplicate. Dotted line represents the cut-off value (48,000 RLU).

We assessed the presence of antibodies against ASP in the plasma from two groups of HIV-1 infected individuals: a group of European patients under ART and a group of African patients who discontinued ARV (Nagot et al., 2016). The group of African patients were pregnant women recruited for the ANRS 12174 clinical trial (Nagot et al., 2016). In this trial, women received zidovudine as prophylaxis for 4–16 weeks before delivery followed by a combination of zidovudine and lamivudine for 7 days after delivery (Table 1). Maternal prophylaxis was stopped at day 7 after patients gave birth, and plasma samples were then regularly collected up to 41 weeks following ARV cessation. The results presented Figure 1 were obtained on plasma samples collected from the African patients 37 weeks after ARV cessation (W37).

**TABLE 1:** Immuno-virological parameters of the patients belonging to the cohort ANRS 12174 (PROMISE-PEP study).

**Patient**	**Ethnical**	**ARV received**	**Duration of**	**Time after ARV**	**Viremia (copies of HIV-1**	**CD4 T cells/**
	**origin**	**during pregnancy**	**ARV (weeks)**	**cessation (weeks)**	**RNA/mL of blood)**	**μL of blood**
P#1	African	ZDV	11	37	1146000	403
P#2	African	ZDV	16	37	609800	536
P#3	African	ZDV	11	37	NA	NA
P#4	African	ZDV	14	37	438900	522
P#5	African	ZDV	10	37	NA	NA
P#6	African	ZDV	8	37	338600	395
P#7	African	ZDV	7	0	17280	NA
				5	NA	NA
				13	2492000	536
				25	NA	NA
				37	NA	NA
				41	1437000	523
P#8	African	ZDV	6	37	92330	468
P#9	African	ZDV	7	0	50320	NA
				13	279400	367
				25	NA	NA
				37	NA	NA
				41	394100	421
P#10	African	ZDV	13	37	NA	NA
P#11	African	ZDV	5	25	NA	NA
P#12	African	ZDV	0	37	1712	728
P#13	African	ZDV	13	37	9069	4057
P#14	African	ZDV	8	37	318700	729
P#15	African	ZDV	14	37	250400	545
P#16	African	ZDV	4	0	Undetectable	NA
				14	NA	NA
				18	21160	477
				25	NA	NA
				37	3931	831
P#17	African	ZDV	11	37	17210	847
P#18	African	ZDV	6	37	1128000	372
P#19	African	ZDV	2	37	NA	NA

*ARV, Antiretroviral agent; ZDV, zidovudine. NA, not available.*

We used two groups of uninfected individuals as a control: a group of breastfeeding women from Africa, and a group of European individuals (Figure 1 and Table 1). Using both control groups, the cut-off for positive anti-ASP response was set-up at 48,000 RLU (see section “Materials and Methods”). In the plasma of patients under ART, we observed a signal similar to that obtained in the plasma of the uninfected individuals. However, we observed a positive signal in 3 of the 19 plasma samples collected from the African patients at W37, indicating the presence of antibodies against ASP in the plasma of these three patients (patients #7, #9, and #16, Figure 1A). We then determined the titer of ASP-specific antibodies in the plasma of patients #7, #9, and #16 at W37 by performing serial dilutions of the plasma samples (Figure 1B). Patients #7 and #9 displayed ASP-antibody titers of 1/200 and 1/100, respectively. In accordance with its higher luminescence signal, the patient #16 showed an ASP-antibody titer of 1/800. Altogether, we detected antibodies against ASP in 3 out of the 19 HIV-1-infected patients (15%) who discontinued ARV, but not in patients under ART.

### Antibody Response to ASP Is Dynamic Over Months

**FIGURE 2:**
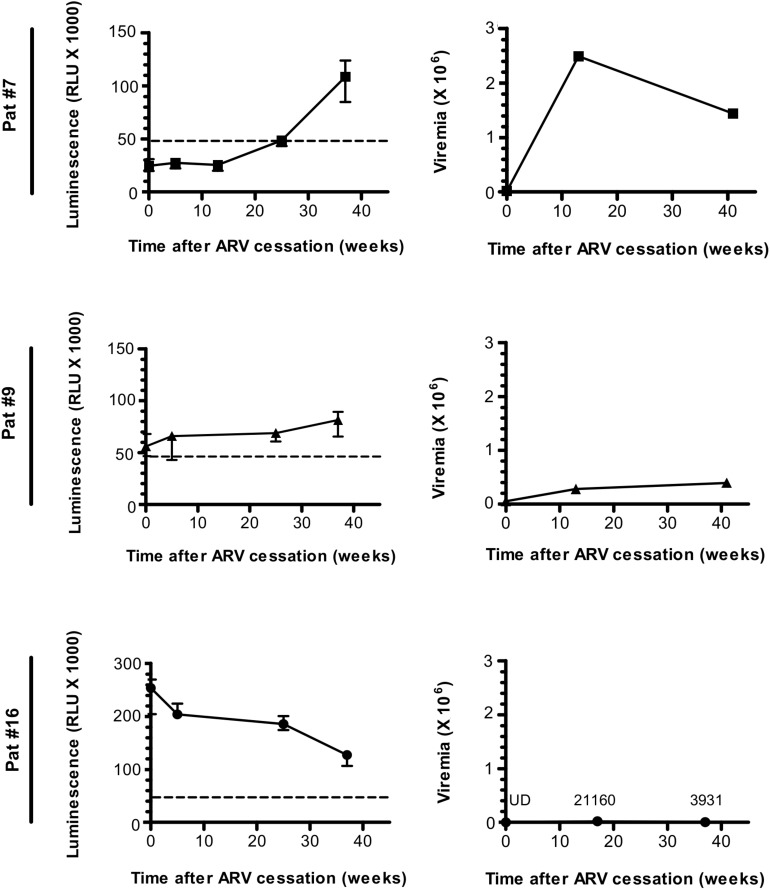
The Antibody response-targeting ASP fluctuates following ARV cessation. LIPS was conducted on the plasma samples collected from Pat #7, Pat #9, and Pat #16 at different weeks after ARV cessation. Signal is expressed as Relative Light Units (RLU). Data shown represent the median (plus min and max values) of one representative experiment performed in triplicates. Values of viremia (number of HIV-1 RNA copies per mL) measured in the context of the ANRS12174 trial are also represented. For Pat #16, values of viremia are indicated at each time point. Dotted line represents the cut-off value (48,000 RLU).

To evaluate the evolution of the antibody response to ASP overtime, we searched for ASP-specific antibodies in the plasma samples of Pat #7, #9, and #16 which were collected at different times from ARV cessation to W37, as previously mentioned (Nagot et al., 2016) (Figure 2 and Table 1). Interestingly, the anti-ASP antibody response fluctuated differently following ARV cessation in the plasma of these three patients. In the plasma of Pat #16, the response to ASP declined overtime, though it remained the highest antibody response to ASP among the three patients, whatever the time points considered. On the contrary, anti-ASP response remained quite stable in the plasma of Pat #9 during the follow-up. For Pat #9 and #16 plasma, antibodies against ASP were detected at the time of ARV cessation, while they only became detectable 25 weeks after ARV cessation in Pat #7. Levels of antibodies to ASP then increased up to 37 weeks post-ARV cessation in Pat #7, concomitantly to a decrease in viral loads. Of note, the plasma of Pat #16 displayed sustained low viremia and increased levels of CD4+ T cells between ARV cessation and W37 (Figure 2 and Table 1), while the level of CD4+ T cells remained stable for Pat #7 and #9 during the follow-up. Overall, the antibody response in the plasma of Pat #7, #9, and #16 to ASP fluctuated after ARV cessation in a patient specific manner.

### Antibodies to ASP Are Not Detected in Patients Under ART

**FIGURE 3:**
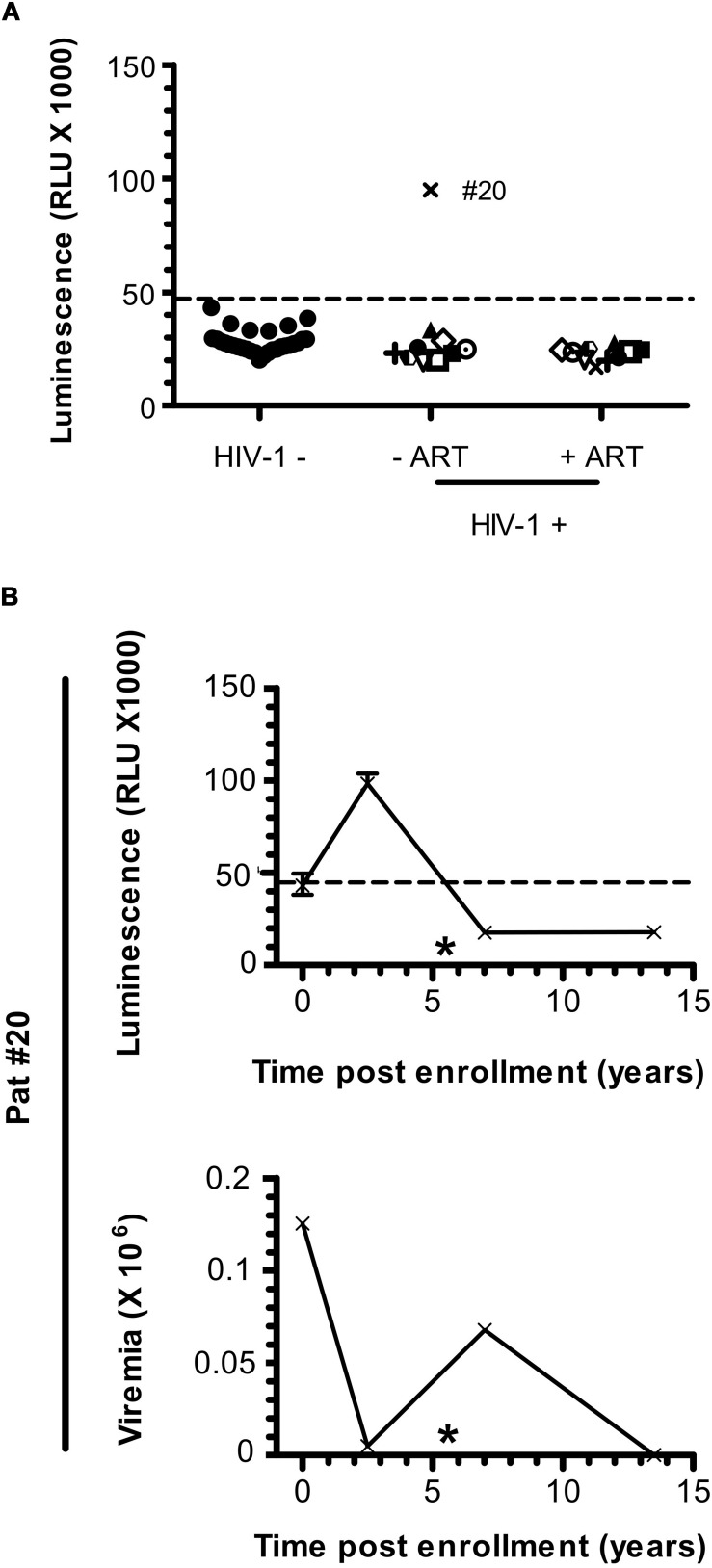
Anti-ASP antibody response is not detected after ART initiation in Pat #20. **(A)** The presence of antibodies against ASP was monitored in the plasma of 10 patients from the SEROCO cohort before and after they initiated ART. Time elapsed between the two time points differ for each patient and goes from 7.5 to 13 years. Each patient is identified by a different symbol. Results obtained with the French seronegative control group (*N* = 20) presented Figure 1 are also shown. Signal is expressed as Relative Light Units (RLU). Data shown are the mean of the median of three independent experiments performed in triplicates. **(B)** The anti-ASP antibody response and viremia overtime were monitored in Pat #20 at four different times post inclusion (at 2.5- and 13.5-years post-inclusion as showed in panel **(A)**, at inclusion and at 7 years post-inclusion). Data shown represent the median (plus min and max values) of one representative experiment performed in triplicates. The star indicates initiation of ART. Values of viremia (number of HIV-1 RNA copies per mL) previously measured in the context of the SEROCO cohort are also represented. Dotted line represents the cut-off value (48,000 RLU).

Patients under ART did not show any specific signal for anti-ASP antibodies (Figure 1A). To further investigate the fluctuations of the antibody response to ASP upon ART initiation, we performed LIPS assay on the plasma of 10 patients from the French ANRS SEROCO cohort (Multicentric Cohort of HIV Positive Patients), before and after they initiated ART (Hubert et al., 2000) (Figure 3A and Table 2). Strikingly, we detected a strong signal in the plasma of 1 of the 10 patients prior to ART (Pat #20 at 2.5 years post-enrollment, i.e., 3 years before ART initiation), but we did not detect any specific signal in the plasma of this same patient 8 years after ART initiation (Table 2). To further characterize the response developed against ASP in Pat #20, we conducted LIPS assay on plasma samples collected at two additional times post-enrollment, respectively 5 years before and 2 years after ART initiation (Figure 3B and Table 2). Interestingly, the loss of antibody response to ASP observed in the plasma of Pat #20 was already observed 2 years after ART initiation and seemed to be sustained for up to 8 years. Besides, antibodies to ASP were barely detectable in this patient at enrollment (5 years before ART), while increased levels were detected 2.5 years post-enrollment (i.e., 3 years before ART). As patients from the SEROCO cohort were infected no more than 1 year prior to their enrollment (Lambotte et al., 2005; Sáez-Cirión et al., 2007), these data suggest that levels of antibodies to ASP kept increasing following the first year of infection in Pat #20. Besides, as observed for Pat #7 (Figure 2), increased levels of anti-ASP antibodies in the plasma of Pat #20 were detected concomitantly to a phase of viremia decline. Altogether, these results suggest that the magnitude of the humoral response to ASP decreases under ART.

**TABLE 2:** Immuno-virological parameters of the patients belonging to the cohort ANRS CO2 SEROCO.

**Patient**	**Ethnical origin**	**Time post-inclusion (years)**	**ART**	**Viremia (copies of HIV-1 RNA/mL of blood)**	**CD4 T cells/μL of blood**
Pat#20	European	0	No	125430	1200
		2,5	No	4931	690
		7	AZT-DDC	67722	139
		13,5	AZT-DDC-InR	Undetectable	58
Pat#21	European	3	No		413
		15	3TC-ABC-NVP		572
Pat#22	European	2,5	No		805
		8,5	3TC-AZT-IDV		171
Pat#23	European	3,5	No		514
		15	AZT-DDI		558
Pat#24	European	2,5	No		720
		10,75	AZT-DDC-SQV		768
Pat#25	European	1,5	No		791
		14,5	ABC-DDI-EFV		649
Pat#26	European	2,5	No		600
		12,5	D4T-NVP-SqR		1087
Pat#27	European	5	No		350
		13	3TC-ABC-AZT		776
Pat#28	European	4,5	No		785
		12	3TC-D4T		681
Pat#29	European	4,5	No		500
		13	3TC-D4T		652

*AZT, Azidothymidine; DDC, Dideoxycytidine; lnR, Lopinavir; 3TC, Lamivudine; ABC, Abacavir; NVP, Nevirapine; IDV, Indinavir; DDI, Didanosine; SQV, Saquinavir; EFV, Efavirenz; SqR, Saquinavir; D4T, Stavudine.*

### Antibodies to ASP Are Not Detected in Aviremic HIV Controllers

**FIGURE 4:**
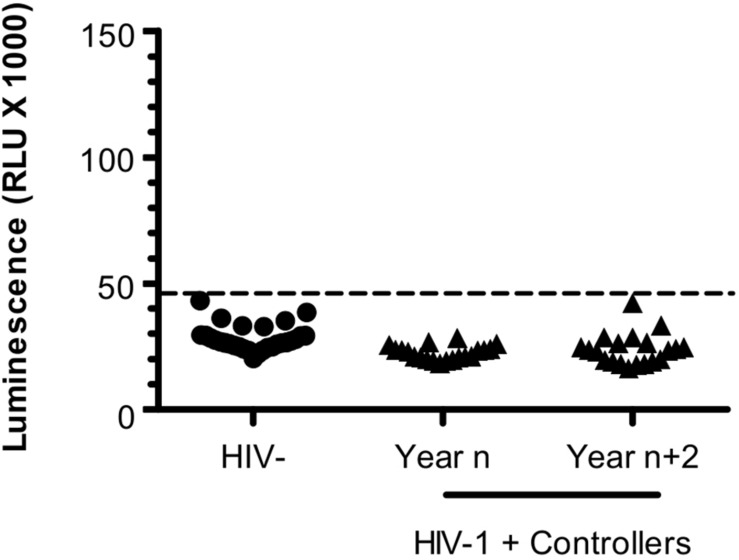
Antibodies targeting ASP are not detected in HIV Controllers. LIPS was conducted on the plasma of 20 HIV-1 controllers collected at two different times -2 years apart; year n/year *n*+2- after their inclusion in the CODEX cohort. Results obtained with the French seronegative control group (*N* = 20) presented Figure 1 are also shown. Signal is expressed as Relative Light Units (RLU). Data shown are the mean of the median of three independent experiments performed in triplicates. Dotted line represents the cut-off value (48,000 RLU).

In plasma samples of Pat #7 and #20, increased levels of anti-ASP antibodies were observed during a phase of viremia decline, which could indicate that antibodies targeting ASP contributed to the immune response elicited against HIV-1. To test this hypothesis, we investigated the presence of ASP-specific antibodies in the plasma of HIV-1 controllers (HIC) enrolled in the French ANRS CODEX cohort (Multicentric cohort of HIV-1 patients with extreme progression profiles; Table 3) (Lambotte et al., 2005; Sáez-Cirión et al., 2007). HIC are patients naturally displaying long-term control of viremia (below 400 HIV RNA copies/mL in the CODEX cohort). As we showed above that the antibody response to ASP fluctuated overtime in patients (Figures 2, 3B), we assessed anti-ASP antibodies in the plasma samples collected from these patients at two different times post-enrollment, 2 years apart (Table 3). As showed Figure 4, only a background signal was observed in the plasma of these patients, indicating that they did not display antibodies to ASP at any of the two time points that we tested. Of note, we cannot totally exclude the possibility that these patients presented anti-ASP antibodies at earlier stages of the infection, but according to our results, ASP-specific antibodies probably did not contribute to set up the control of viremia in these patients.

**TABLE 3:** Immuno-virological parameters of the patients belonging to the cohort ANRS HIV Controller CO21 CODEX.

**Patient**	**Ethnical**	**Time post-**	**Viremia**	**CD4 T**
	**origin**	**inclusion**	**(copies of HIV-1**	**cells/μL**
		**(years)**	**RNA/mL of blood)**	**of blood**
Pat#30	European	1	<20	1238
		3	<20	1917
Pat#31	European	0	<50	809
		2	<40	874
Pat#32	European	0	<10	790
		2	<10	1153
Pat#33	African (Maghreb)	4	<40	1216
		6	<40	1320
Pat#34	European	2	41	478
		4	<40	554
Pat#35	European	3	<40	644
		5	42	539
Pat#36	European	1	<20	676
		3	<20	628
Pat#37	European	3	<40	452
		6	<40	630
Pat#38	European	0	<20	627
		2	<20	662
Pat#39	European	0	<40	1224
		2	<40	1482
Pat#40	African (Maghreb)	0	<40	421
		2	<40	666
Pat#41	European	1	<20	712
		3	23	979
Pat#42	European	2	<20	1382
		4	<20	1509
Pat#43	European	2	<40	966
		4	<40	851
Pat#44	European	1	<40	570
		3	<30	600
Pat#45	European	2	<20	780
		4	<20	1740
Pat#46	European	0	<20	989
		2	<20	761
Pat#47	European	0	<20	584
		2	<20	693
Pat#48	European	3	<20	704
		5	<40	900
Pat#49	European	0	<40	735
		2	<35	836

### Antibodies Target the 26–141 Region of ASP

**FIGURE 5:**
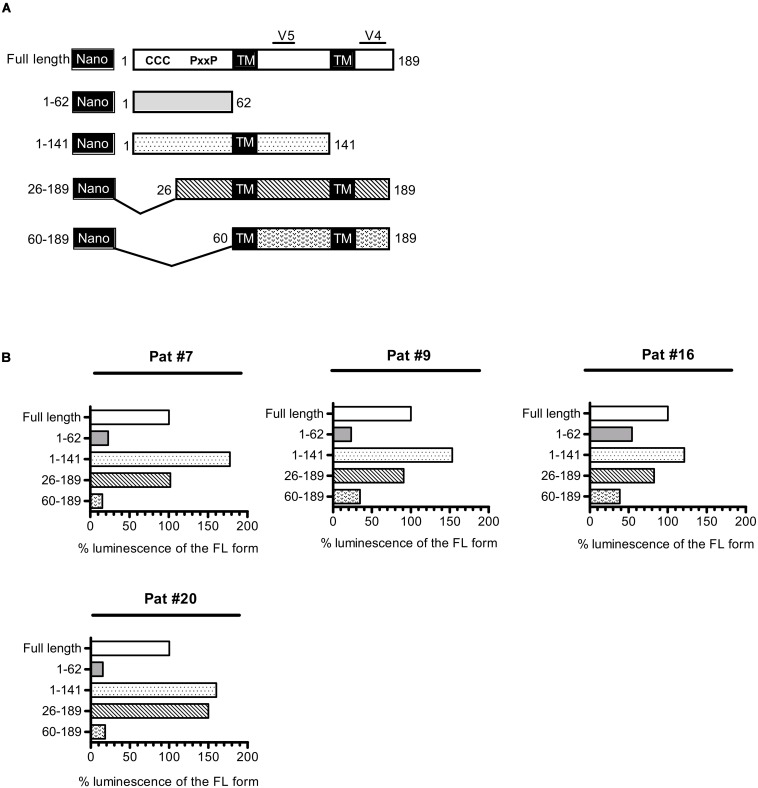
Patient antibodies target the 26–141 core region of ASP encompassing the V5 and prolin-rich motifs. **(A)** Scheme of nano-ASP mutants: CCC, cysteine triplets; PxxP, prolin-rich motif; TM, potential transmembrane domain; V4/V5, highly variable motifs 4 and 5. The numbers indicate amino acid positions in ASP. **(B)** LIPS was conducted with nano-ASP mutants on the plasma collected from Pat #7, #9, #16 (collected 37 weeks after ARV cessation), and #20 (collected 2.5 years after enrollment). For each mutant, the signal was normalized by substracting the signal obtained with the nanoluciferase alone for the same plasma sample. The resulting normalized luminescence is expressed as the percentage of the signal obtained with the full-length form of nano-ASP. Data shown represent the median of the triplicates obtained from one representative experiment.

As a consequence of its overlapping position to *env* in the proviral genome of HIV-1, *asp* encodes for a protein that possesses two highly variable regions (V4 and V5, Figure 5) located at its C-terminal extremity. Conversely, the N-terminal extremity of ASP displays several well-conserved domains: two cysteine triplets and a prolin-rich motif. To delineate the domains of ASP which are targeted by antibodies, we conducted LIPS on the plasma collected from Pat #7, #9, #16, and #20 with nano-ASP mutants deleted from different domains of ASP (Figure 5 and Supplementary Figure S2). The deletion of the cysteine triplets (26–189 mutant) and of the C-terminal region (1–141 mutant) encompassing the highly variable motif V4 of ASP did not decrease the signal in any of the plasma tested, indicating that none of these regions are targeted by the antibodies that are present in the plasma of Pat #7, #9, #16, and #20. On the contrary, the signal was either reduced (Pat #9 and #16) or totally suppressed (Pat #7 and #20) with the ASP 60–189 mutant lacking the prolin-rich motif. In all the patients, a strong decline was observed when using the prolin-rich motif as antigen (1–62 mutant), although the decline was less important for Pat #16 than for the three other patients. These results suggest that both the 26–60 region encompassing the PxxP motif and the 60–141 core region of ASP encompassing the highly variable motif V5 are involved in the main epitope of ASP, either directly or indirectly by allowing the proper folding of the epitope.

## Discussion

In our study, we developed a specific and quantitative luminescent assay to assess the presence of antibodies directed against ASP in the plasma of HIV-1-infected patients. Using this approach, we found anti-ASP antibodies in patients who discontinued ARV (3 of 19) and in ART-naive patients (1 of 10). By contrast, antibodies to ASP were detected neither in aviremic patients under ART nor in ART-naive aviremic HIV–1 controllers. The analysis of ASP-targeted domains showed that both the conserved prolin-rich motif and the core region (60–141) encompassing the highly variable motif V5 are involved in ASP recognition by antibodies. So far, the only variation of ASP that has been specifically associated with a viral subtype (HIV-1 subtype A) is a N-terminal deletion (residues 1–26) (Cassan et al., 2016). Besides, the C-terminal domain contains most of the inter- and intra-individual variations (Cassan et al., 2016). Neither the N-terminal nor the C-terminal extremities of ASP seemed to be targeted by antibodies, which thus appear to be dispensable to elicit anti-ASP antibody response. However, we cannot exclude that, in our investigation, the targeting of the variable C-terminal region of ASP might have been underestimated by the use of an ASP antigen (derived from BRU/LAV) whose sequence might differ from the ones found in patients.

In our study, anti-ASP antibodies were detected in 10–15% of the HIV-1-infected individuals, which seems less than previously suggested by the study of C. Vaquero, a least for patients in advanced stages of infection (Vanhée-Brossollet et al., 1995). However, humoral responses to other auxiliary and regulatory proteins of HIV-1 in infected subjects ranged from 12% (Tat; Rezza et al., 2005) to 25–26% (Vif and Vpr; Reiss et al., 1990; O’Neil et al., 1997), and up to 43% (Vpu; Reiss et al., 1990). The frequency of anti-ASP antibody response is also consistent with previous studies showing humoral responses against HBZ in 10% of the HTLV-1 infected subjects (Enose-Akahata et al., 2013; Shiohama et al., 2016). The CD8+ T cell response targeting ASP was described in approximately 30% of the patients (Berger et al., 2015; Bet et al., 2015). This higher frequency might be inferred to the targeting by CD8+ T cells of highly conserved linear epitopes from intracellular antigens, contrary to antibodies which target extracellular antigens. Humoral responses to HIV-1 Gag, Pol and Env proteins were described to be dependent of HIV-1 replication and to strongly decline during ART (Voltersvik et al., 2003; Killian et al., 2006; Zhang et al., 2017; Delagreverie et al., 2019). In line with these results, we did not detect any antibodies to ASP in aviremic patients under ART. In addition, antibodies to ASP were no longer detected in Pat #20 at two different times after ART initiation, further suggesting that the prolonged aviremia under ART lead to a decrease of antibody responses to ASP. However, for Pat #9 and #16 who presented antibodies to ASP at the earliest time point tested after ARV cessation, we can speculate a persistence of this response regarding the short duration of their ARV (7 and 4 weeks, respectively). As suggested by the lack of detection of antibodies targeting ASP in the plasma of HIV-1 controllers in our study, a detectable level of viral replication might be needed to induce a humoral response to ASP. Interestingly, ASP was recently described at the membrane of viral particles released by chronically infected cells following their *in vitro* reactivation (Affram et al., 2019). If ASP is indeed present at the surface of viral particles *in vivo*, it might partially explain why we did not detect antibodies against ASP in patients displaying very low or undetectable viral loads. However, this hypothesis is challenged by the fact that we observed a peak of antibodies to ASP concomitantly to a phase of viremia decline in Pat #7, and during a phase of low viremia in Pat #20. As our results suggest that anti-ASP response is dynamic overtime, a deeper study of the relationship between viremia and the antibody response against ASP would require the analysis of these parameters in plasma sampled from patients at shorter temporal intervals.

This could also indicate that the *in vivo* production of ASP occurs when viral production decreases, contrary to what was suggested by the study of Affram et al. (2019), but in line with a previous study which suggested that ASP is rather expressed during the chronical phase than during the acute phase of the disease (Bet et al., 2015). Besides, we recorded an increase in ASP-specific antibody titers during the 2.5 years following enrollment in Pat #20, further suggesting that antibody response to ASP is developed after acute infection. Though a recent study reported the concomitant expression of sense and antisense transcripts in infected cells (Mancarella et al., 2019), several studies conducted by our group and by others showed that antisense transcription preferentially occurs in non-producing infected cells (Landry et al., 2007; Kobayashi-Ishihara et al., 2012; Laverdure et al., 2012; Saayman et al., 2014; Zapata et al., 2017). This consideration is of outstanding interest as several studies previously proposed antisense transcription as a common feature of viral latency (Landry et al., 2007; Kobayashi-Ishihara et al., 2012; Laverdure et al., 2012; Saayman et al., 2014; Zapata et al., 2017). Altogether, we developed a specific and quantitative assay that allows us to evaluate anti-ASP antibodies in the plasma of HIV-1-infected patients. Our results provide the first confirmation of the study conducted in 1995 by C. Vaquero’s group (Vanhée-Brossollet et al., 1995) and describe individual variations in ASP-specific antibody titers during the infection course. Though observed on a small number of subjects, the results of our pilot evaluation point the importance to study ASP and its antibody response during the different stages of HIV-1 infection. In the future, it would be of great interest to increase the number of serum samples (from HIV-1 negative and positive individuals) tested and to study the long-term ASP-specific antibody response in untreated, chronically infected subjects and to assess its potential impact on disease outcomes.

## Data Availability Statement

The raw data supporting the conclusion of this article will be made available by the authors, without undue reservation, to any qualified researcher.

## Ethics Statement

The studies involving human participants were reviewed and approved by the ANRS 12174 trial: subjects were HIV-1-infected breastfeeding women from Burkina Faso whose infants were enrolled in the PROMISE-PEP trial. All mothers received AZT during pregnancy and labor but they were not eligible for ART post-partum according to national recommendations at the period the trial was conducted (above 350 CD4 cell/μL of blood) (Nagot et al., 2016). Written informed consents were obtained from the mother or the legal representative prior to enrollment (NCT00640263). Plasma samples were screened at week 38 post-partum and at intermediate time points when necessary. The study was conducted according to the sponsor (Agence Nationale de Recherche sur le Sida et les hépatites virales; ANRS) ethic charter, Good Clinical Practices and the principles of the Helsinki declaration. The protocol was approved by the National Ethics Committee (n° 2008-0039). ANRS CO2 SEROCO cohort: subjects, enrolled from 1988 to 1995 (i) were infected no more than 12 months prior to enrollment and (ii) were free of AIDS related diseases at inclusion (Hubert et al., 2000). No ART were received before inclusion and during the study until 1996. A follow-up was performed every 6 months until 2009. A written informed consent was obtained from patients, in line with the guidelines formulated by local ethical committees. ANRS HIV Controller CO21 CODEX cohort: Enrolled subjects met the following criteria: (i) naive for ART, (ii) seropositive for at least 5 years before enrollment, and (iii) with a viral load below 400 HIV-1 RNA copies/mL in the five consecutive measurements before inclusion (Lambotte et al., 2005; Sáez-Cirión et al., 2007). Follow-up was performed at least once a year for 6 years. All patients gave a written informed consent. The C021 CODEX cohort was approved by the Ethics Review Committee of Ile de France VII and performed in compliance with the tenets of the Declaration of Helsinki. Patients under ART: we used plasma isolated from total blood samples collected from 20 patients under ART during their follow-up at the University Hospital of Montpellier. Subjects enrolled in the cohort provided written informed consent. The study was then approved by the Institutional Ethics Committee (RCB n° 2011-A01566-35, AC-2011-1405). Uninfected donors: plasma samples were collected from French seronegative blood donors (Etablissement Français du Sang) and from breastfeeding, seronegative women from Zambia. The study was approved by the Excellency in Research Ethics and Science Converge Institutional Review Board (00005948 IRB number) in Lusaka, Zambia. The patients/participants provided their written informed consent to participate in this study.

## Author Contributions

AG, J-MM, PV, and J-PM designed the study. JS performed all experiments with assistance of NC and AG. JS, NC, and AG analyzed the data and wrote the manuscript. J-PM, ET, and PV provided samples from the PROMISE-PEP cohort and the University Hospital of Montpellier. FB and LM provided samples from the SEROCO cohort. CL and OL provided samples from the CODEX cohort.

## Conflict of Interest

The authors declare that the research was conducted in the absence of any commercial or financial relationships that could be construed as a potential conflict of interest.

## References

[B1] Affram Y. Zapata J. C. Gholizadeh Z. Tolbert W. D. Zhou W. Iglesias-Ussel M. D. (2019). The HIV-1 antisense protein ASP is a transmembrane protein of the cell surface and an integral protein of the viral envelope. *J. Virol.* 93:e00574-19. 10.1128/JVI.00574-19 31434734 PMC6803264

[B2] Arpin-André C. Laverdure S. Barbeau B. Gross A. Mesnard J.-M. (2014). Construction of a reporter vector for analysis of bidirectional transcriptional activity of retrovirus LTR. *Plasmid* 74 45–51. 10.1016/j.plasmid.2014.06.001 24971710

[B3] Berger C. T. Llano A. Carlson J. M. Brumme Z. L. Brockman M. A. Cedeño S. (2015). Immune screening identifies novel T cell targets encoded by antisense reading frames of HIV-1. *J. Virol.* 89 4015–4019. 10.1128/JVI.03435-3414 25589651 PMC4403399

[B4] Bet A. Maze E. Bansal A. Sterrett S. Gross A. Graff-Dubois S. (2015). The HIV-1 Antisense Protein (ASP) induces CD8 T cell responses during chronic infection. *Retrovirology* 12:15. 10.1186/s12977-015-0135-y 25809376 PMC4335690

[B5] Briquet S. Vaquero C. (2002). Immunolocalization studies of an antisense protein in HIV-1-infected cells and viral particles. *Virology* 292 177–184. 10.1006/viro.2001.1224 11878921

[B6] Burbelo P. D. Bayat A. Rhodes C. S. Hoh R. Martin J. N. Fromentin R. (2014). HIV antibody characterization as a method to quantify reservoir size during curative interventions. *J. Infect. Dis.* 209 1613–1617. 10.1093/infdis/jit667 24286982 PMC3997576

[B7] Burbelo P. D. Lebovitz E. E. Notkins A. L. (2015). Luciferase immunoprecipitation systems for measuring antibodies in autoimmune and infectious diseases. *Transl Res.* 165 325–335. 10.1016/j.trsl.2014.08.006 25241936 PMC4306608

[B8] Cassan E. Arigon-Chifolleau A.-M. Mesnard J.-M. Gross A. Gascuel O. (2016). Concomitant emergence of the antisense protein gene of HIV-1 and of the pandemic. *Proc. Natl. Acad. Sci. U.S.A.* 113 11537–11542. 10.1073/pnas.1605739113 27681623 PMC5068275

[B9] Clerc I. Laverdure S. Torresilla C. Landry S. Borel S. Vargas A. (2011). Polarized expression of the membrane ASP protein derived from HIV-1 antisense transcription in T cells. *Retrovirology* 8:74. 10.1186/1742-4690-8-74 21929758 PMC3182985

[B10] Delagreverie H. M. Grude M. Lambert-Niclot S. Nere M.-L. Jadand C. Leport C. (2019). Anti-gp41 antibody levels reflect HIV viral suppression and cellular reservoir in long-term antiretroviral-treated trial participants. *J. Antimicrob. Chemother.* 74 1389–1394. 10.1093/jac/dkz004 30690509

[B11] Enose-Akahata Y. Abrams A. Massoud R. Bialuk I. Johnson K. R. Green P. L. (2013). Humoral immune response to HTLV-1 basic leucine zipper factor (HBZ) in HTLV-1-infected individuals. *Retrovirology* 10:19. 10.1186/1742-4690-10-19 23405908 PMC3584941

[B12] Gaudray G. Gachon F. Basbous J. Biard-Piechaczyk M. Devaux C. Mesnard J.-M. (2002). The complementary strand of the human T-cell leukemia virus type 1 RNA genome encodes a bZIP transcription factor that down-regulates viral transcription. *J. Virol.* 76 12813–12822. 10.1128/jvi.76.24.12813-12822.2002 12438606 PMC136662

[B13] Hubert J. B. Burgard M. Dussaix E. Tamalet C. Deveau C. Le Chenadec J. (2000). Natural history of serum HIV-1 RNA levels in 330 patients with a known date of infection. The SEROCO Study Group. *AIDS* 14 123–131. 10.1097/00002030-200001280-00007 10708282

[B14] Killian M. S. Norris P. J. Rawal B. D. Lebedeva M. Hecht F. M. Levy J. A. (2006). The effects of early antiretroviral therapy and its discontinuation on the HIV-specific antibody response. *AIDS Res. Hum. Retroviruses* 22 640–647. 10.1089/aid.2006.22.640 16831088

[B15] Kobayashi-Ishihara M. Yamagishi M. Hara T. Matsuda Y. Takahashi R. Miyake A. (2012). HIV-1-encoded antisense RNA suppresses viral replication for a prolonged period. *Retrovirology* 9:38. 10.1186/1742-4690-9-38 22569184 PMC3410806

[B16] Lambotte O. Boufassa F. Madec Y. Nguyen A. Goujard C. Meyer L. (2005). HIV controllers: a homogeneous group of HIV-1–infected patients with spontaneous control of viral replication. *Clin. Infect. Dis.* 41 1053–1056. 10.1086/433188 16142675

[B17] Landry S. Halin M. Lefort S. Audet B. Vaquero C. Mesnard J.-M. (2007). Detection, characterization and regulation of antisense transcripts in HIV-1. *Retrovirology* 4:71. 10.1186/1742-4690-4-71 17910760 PMC2099442

[B18] Laverdure S. Gross A. Arpin-André C. Clerc I. Beaumelle B. Barbeau B. (2012). HIV-1 antisense transcription is preferentially activated in primary monocyte-derived cells. *J. Virol.* 86 13785–13789. 10.1128/JVI.01723-1712 23035216 PMC3503093

[B19] Mancarella A. Procopio F. A. Achsel T. De Crignis E. Foley B. T. Corradin G. (2019). Detection of antisense protein (ASP) RNA transcripts in individuals infected with human immunodeficiency virus type 1 (HIV-1). *J. Gen. Virol.* 100 863–876. 10.1099/jgv.0.001244 30896385

[B20] Michael N. L. Vahey M. T. d’Arcy L. Ehrenberg P. K. Mosca J. D. Rappaport J. (1994). Negative-strand RNA transcripts are produced in human immunodeficiency virus type 1-infected cells and patients by a novel promoter downregulated by Tat. *J. Virol.* 68 979–987. 10.1128/jvi.68.2.979-987.1994 8289399 PMC236536

[B21] Miller R. H. (1988). Human immunodeficiency virus may encode a novel protein on the genomic DNA plus strand. *Science* 239 1420–1422. 10.1126/science.3347840 3347840

[B22] Nagot N. Kankasa C. Tumwine J. K. Meda N. Hofmeyr G. J. Vallo R. (2016). Extended pre-exposure prophylaxis with lopinavir–ritonavir versus lamivudine to prevent HIV-1 transmission through breastfeeding up to 50 weeks in infants in Africa (ANRS 12174): a randomised controlled trial. *Lancet* 387 566–573. 26603917 10.1016/S0140-6736(15)00984-8

[B23] O’Neil C. Lee D. Clewley G. Johnson M. A. Emery V. C. (1997). Prevalence of anti-vif antibodies in HIV-1 infected individuals assessed using recombinant baculovirus expressed vif protein. *J. Med. Virol.* 51 139–144. 9139075 10.1002/(sici)1096-9071(199703)51:3<139::aid-jmv1>3.0.co;2-7

[B24] Reiss P. Lange J. M. de Ronde A. de Wolf F. Dekker J. Danner S. A. (1990). Antibody response to viral proteins U (vpu) and R (vpr) in HIV-1-infected individuals. *J. Acquir. Immune Defic. Syndr.* 3 115–122. 2136912

[B25] Rezza G. Fiorelli V. Dorrucci M. Ciccozzi M. Tripiciano A. Scoglio A. (2005). The presence of anti-Tat antibodies is predictive of long-term nonprogression to AIDS or severe immunodeficiency: findings in a cohort of HIV-1 seroconverters. *J. Infect. Dis.* 191 1321–1324. 10.1086/428909 15776379

[B26] Saayman S. Ackley A. Turner A.-M. W. Famiglietti M. Bosque A. Clemson M. (2014). An HIV-Encoded Antisense Long Noncoding RNA Epigenetically Regulates Viral Transcription. *Molecular Therapy* 22 1164–1175. 10.1038/mt.2014.29 24576854 PMC4048891

[B27] Sáez-Cirión A. Lacabaratz C. Lambotte O. Versmisse P. Urrutia A. Boufassa F. (2007). HIV controllers exhibit potent CD8 T cell capacity to suppress HIV infection ex vivo and peculiar cytotoxic T lymphocyte activation phenotype. *Proc. Natl. Acad. Sci. U.S.A.* 104 6776–6781. 10.1073/pnas.0611244104 17428922 PMC1851664

[B28] Shiohama Y. Naito T. Matsuzaki T. Tanaka R. Tomoyose T. Takashima H. (2016). Absolute quantification of HTLV-1 basic leucine zipper factor (HBZ) protein and its plasma antibody in HTLV-1 infected individuals with different clinical status. *Retrovirology* 13:29. 10.1186/s12977-016-0263-z 27117327 PMC4847349

[B29] Torresilla C. Larocque É Landry S. Halin M. Coulombe Y. Masson J.-Y. (2013). Detection of the HIV-1 minus-strand-encoded antisense protein and its association with autophagy. *J. Virol.* 87 5089–5105. 10.1128/JVI.00225-213 23427159 PMC3624327

[B30] Vanhée-Brossollet C. Thoreau H. Serpente N. D’Auriol L. Lévy J.-P. Vaquero C. (1995). A natural antisense RNA derived from the HIV-1 env gene encodes a protein which is recognized by circulating antibodies of HIV+ individuals. *Virology* 206 196–202. 7831774 10.1016/s0042-6822(95)80034-4

[B31] Voltersvik P. Albrektsen G. Ulvestad E. Dyrhol-Riise A. M. Sørensen B. Asjö B. (2003). Changes in immunoglobulin isotypes and immunoglobulin G (IgG) subclasses during highly active antiretroviral therapy: anti-p24 IgG1 closely parallels the biphasic decline in plasma viremia. *J. Acquir. Immune Defic. Syndr.* 34 358–367. 14615653 10.1097/00126334-200312010-00002

[B32] Zapata J. C. Campilongo F. Barclay R. A. DeMarino C. Iglesias-Ussel M. D. Kashanchi F. (2017). The Human Immunodeficiency Virus 1 ASP RNA promotes viral latency by recruiting the Polycomb Repressor Complex 2 and promoting nucleosome assembly. *Virology* 506 34–44. 10.1016/j.virol.2017.03.002 28340355 PMC5505171

[B33] Zhang W. Morshed M. M. Noyan K. Russom A. Sönnerborg A. Neogi U. (2017). Quantitative humoral profiling of the HIV-1 proteome in elite controllers and patients with very long-term efficient antiretroviral therapy. *Sci. Rep.* 7:666. 28386076 10.1038/s41598-017-00759-8 PMC5429677

