# Supplementary material for: A Pilot Study of the Humoral Response Against the AntiSense Protein (ASP) in HIV-1-Infected Patients
Source: Front Microbiol. 2020 Jan 24;11:20. doi: 10.3389/fmicb.2020.00020 (PMC7025555; doi:10.3389/fmicb.2020.00020)
Supplement: Supplementary file 1 [file Data_Sheet_1.PDF]

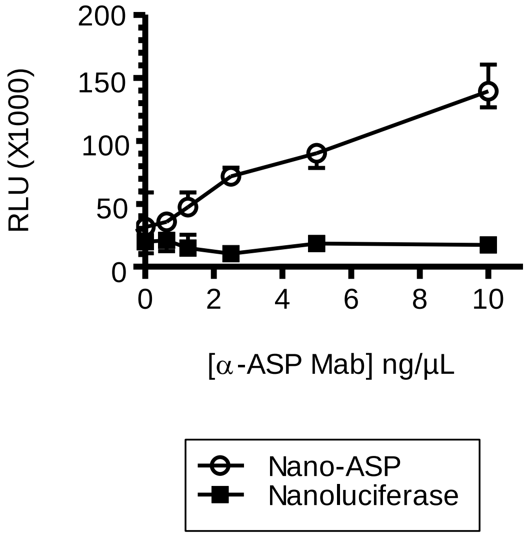

Supplementary Figure 1 : LIPS assay was performed with nano-ASP FL or nanoluciferase alone (negative control) using different concentrations of a monoclonal antibody (Mab) targeting the 47-62 residues of ASP (10, 5, 2.5, 1.25 and 0.625 ng/µL). The dilutions of the Mab were performed in the plasma of uninfected donors diluted 10 fold in LIPS reaction buffer.
