# Supplementary material for: A Pilot Study of the Humoral Response Against the AntiSense Protein (ASP) in HIV-1-Infected Patients
Source: Front Microbiol. 2020 Jan 24;11:20. doi: 10.3389/fmicb.2020.00020 (PMC7025555; doi:10.3389/fmicb.2020.00020)
Supplement: Supplementary file 2 [file Data_Sheet_2.PDF]

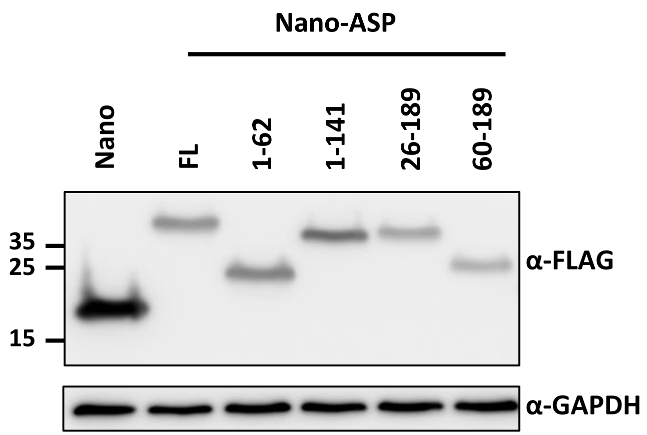

Supplementary Figure 2 : Expression of nanoluciferase, nano-ASP FL (full-length) and nano-ASP mutants in HEK 293 T cells. The numbers indicate the amino acid residues of ASP expressed by each mutant. HEK cells were lysed 48h post-transfection.
The amount of cell extract loaded on the gel was normalized according to total protein content.
